# Metabolic communication in tumors: a new layer of immunoregulation for immune evasion

**DOI:** 10.1186/s40425-016-0109-1

**Published:** 2016-02-16

**Authors:** Ping-Chih Ho, Pu-Ste Liu

**Affiliations:** Department of Fundamental Oncology, University of Lausanne, Lausanne, Switzerland; Ludwig Center for Cancer Research, University of Lausanne, Lausanne, Switzerland

**Keywords:** Immunometabolism, Immune evasion, Immunotherapy, Cancer metabolism, Warburg glycolysis

## Abstract

The success of cancer immunotherapy reveals the power of host immunity on killing cancer cells and the feasibility to unleash restraints of anti-tumor immunity. However, the immunosuppressive tumor microenvironment and low immunogenicity of cancer cells restrict the therapeutic efficacy of cancer immunotherapies in a small fraction of patients. Therefore deciphering the underlying mechanisms promoting the generation of an immunosuppressive tumor microenvironment is direly needed to better harness host anti-tumor immunity. Early works revealed that deregulated metabolic activities in cancer cells support unrestricted proliferation and survival by producing macromolecules. Intriguingly, recent studies uncovered that metabolic switch in immune and endothelial cells modulate cellular activities and contribute to the progression of several diseases, including cancers. Herein, we review the progress on immunometabolic regulations on fine-tuning activities of immune cells and discuss how metabolic communication between cancer and infiltrating immune cells contributes to cancer immune evasion. Moreover, we would like to discuss how we might exploit this knowledge to improve current immunotherapies and the unresolved issues in this field.

## Background

With more than 8 million cancer related deaths, cancer is clearly a major health burden worldwide. Although radiotherapy and chemotherapy elicit strong response rates in the majority of cancer patients, metastatic diseases are problematic to control via these conventional interventions and cures remain scarce. A burgeoning field in oncology and immunology is the ability to eradicate cancer cells by rejuvenating the tumoricidal functions of tumor-reactive immune cells, predominantly T cells. Cancer immunotherapy provides oncologists with a new weapon among existing cancer treatments, which is demonstrated by the recent developments of checkpoint blockade, adoptive cell transfer (ACT) and cancer vaccines. However, the benefit of cancer immunotherapy is currently compromised by the immunosuppressive tumor microenvironment [1, 2]. While the importance of the immunosuppressive tumor microenvironment on dampening the anti-tumor immunity is appreciated, the mechanism by which cancer cells instruct the development of the immunosuppressive microenvironment remains unclear.

Over the past two decades significant understanding has been gained in cancer cell-immune system interactions. Additionally, how the unrestricted proliferation and survival of cancer cells is sustained via the deregulation of cellular energetic pathways has been expounded upon [3, 4]. However, whether the abnormal metabolic activities of cancer cells influence the nutrient states of the tumor microenvironment or the metabolic fitness of neighboring stromal and immune cells remains elusive. Similar to cancer cells, upon stimuli recognition lymphocytes engage metabolic reprogramming to drive their activation and differentiation [5–7]. Therefore, cancer and immune cells share similarities of utilizing nutrients and engaging metabolic regulation to sustain proliferation and survival. This raises an intriguing possibility that “metabolic competition” within the tumor microenvironment may allow cancer cells to effectively suppress anti-tumor immunity. In this review, we focus on how immunometabolic regulations fine-tune the activation and anti-tumor responses of T cells and myeloid cells. Moreover, we discuss the burgeoning idea that metabolic communication and competition within the tumor microenvironment may support the formation of the immunosuppressive tumor microenvironment. Finally, we summarize unresolved issues in this filed and discuss how these issues impact the development of cancer immunotherapy.

## Reviews

### The immunosuppressive tumor microenvironment: a challenge for improving cancer immunotherapies

Progress on exploiting the host anti-tumor immunity to combat established and aggressive tumors presents promising effects, including on two of the most deadly forms of cancer: melanomas and pancreatic cancers. However, there are three major hurdles impeding the affect of host anti-tumor immunity and cancer immunotherapy: 1) low number of tumor antigen-specific T cells due to clonal deletion; 2) poor activation of innate immune cells and accumulation of tolerigenic antigen-presenting cells in the tumor microenvironment; 3) formation of an immunosuppressive tumor microenvironment [8]. Cancer vaccines and T cell-based treatment, such as adoptive cell transfer (ACT) and chimeric-antigen receptor (CAR) T-cells, have overcome the first hurdle and produced remarkable results in several tumors. However, the therapeutic efficacy of these treatments remains unsatisfactory due to the incapability to fully cultivate anti-tumor responses in the immunosuppressive tumor microenvironment [9]. Of note, the Speiser’s group demonstrated that tumor-reactive T cells, upon migrating into tumors, lose their effector functions and increase the expression of co-inhibitory receptors compared to circulating cells [10]. Intriguingly, similar findings have also been shown in murine tumor models. Furthermore, short term *in vitro* culture is able to restore both tumoricidal function and cytokine production in those tumor infiltrating T cells [11]. Taken together, these findings suggest that the tumor microenvironment provides local restraints that abolish the anti-tumor responses of infiltrating T cells. Further investigations uncovered two major underlying mechanisms that disarm anti-tumor immunity in the tumor microenvironment; 1) the accumulation in tumors of immunomodulatory cells, including M2-like macrophages (MΦs), immature dendritic cells, regulatory T cells (Tregs), and myeloid derived suppressor cells (MDSCs), diminishes T cell anti-tumor immune responses through cell-cell contact and cytokine milieu [1, 2]; 2) expression of PD-1 receptor ligands (PD-L1/PD-L2) and reduced expression of tumor antigens and major histocompatibility complex (MHC) in cancer cells [1]. These findings led to the development of anti-CTLA-4 monoclonal antibody treatment, Treg depletion therapy and checkpoint blockade, including PD-1 and PD-L1/L2 inhibition [1, 12, 13].

### Metabolic regulation of T cell anti-tumor responses

#### Metabolic reprogramming guides T cell activation and differentiation

**Fig. 1:**
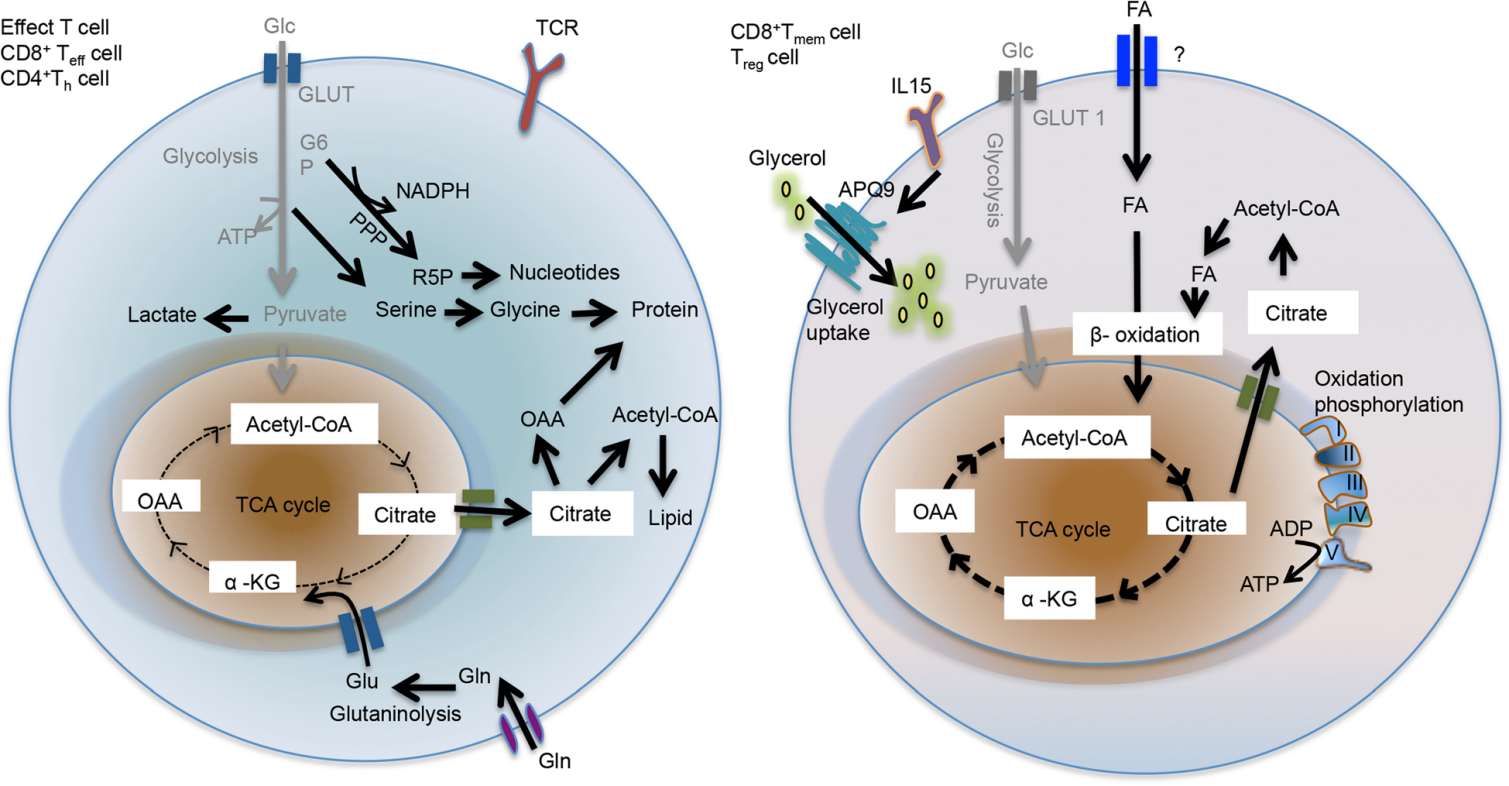
The metabolic profile of activated, effector and memory T cells. CD8^+^ T_mem_ cell and T_reg_ cell rely on FAO and OXPHOS for survival and to support function. In addition, T_mem_ cells acquire glycerol by AQP9 for triglyceride synthesis and then promote T_mem_ cell proliferation and survival (left panel). In right panel, activated effector T cells (Effect T cells, CD8^+^T_eff_ cell and CD4^+^T_h_ cells) use aerobic glycolysis and FAS for proliferation. In activated effector T cell, most pyruvate become lactate and minority of pyruvate enters TCA cycle. However, PPP yields both nucleotide and NADPH for FAS. Glutaminolysis is also important for supplement of TCA cycle intermediates for biosynthesis

Upon receiving T cell receptor (TCR) and co-stimulatory signals, T cells engage in growth, expansion, and ultimately, differentiation into different cytotoxic, Tregs and helper T cells (Th cells; i.e., Th1, Th2 and Th17) [14, 15]. In addition to these defined signaling cascades, changes in metabolic activity has been shown to intimately support T cell differentiation and effector functions [5]. Naïve T cells rely on oxidative phosphorylation (OXOPHOS) to maintain energy demand; in contrast, activated T cells engage aerobic glycolysis consuming massive amount of glucose [16, 17]. Blocking glycolysis or eliminating glucose uptake during T cell activation period diminishes T cell activation, Th cell differentiation, as well as the generation of effector cytokines, including IFNγ, IL-2, and IL-17a in Th1 cells [5, 18]. Conversly, enhancing glycolytic flux in T cells by overexpressing the glucose transporter 1 (GLUT1) escalates inflammatory responses in mice [17]. In contrast to cytotoxic and effector Th cells, Tregs and memory CD8+ T cells rely on OXOPHOS and fatty acid oxidation (FAO) to support their survival and differentiation. Blocking mitochondrial transport of long-chain fatty acids, a rate limiting step of FAO, with carnitine palmitoyltransferase 1A (CPT1a) inhibitors in Tregs suppresses their activity and survival [19]. Mammalian target of rapamycin (mTOR) and AMP-activated protein kinase (AMPK) are, mechanistically, the central energy-monitoring system in cells. Disruption of mTOR and AMPK activity is the critical signaling event that integrates metabolic activity with cell activation and differentiation in T cells. Impairment of glycolytic activity in T cells suppresses mTOR, but boosts AMPK activities, by altering the cellular ratio of AMP/ATP. This leads to a failure to switch T cell metabolism from catabolism into anabolism, affecting amino acid uptake and *de novo* synthesis of amino acids, nucleotides, and fatty acids [5, 18] (Fig. 1). Supporting this, T cell specific mTOR deletion elevates AMPK activity suppressing effector T cell differentiation; but Treg generation from these T cells remains intact. Furthermore, activating AMPK promotes Treg formation in both *in vitro* and *in vitro* assays [19, 20]. In addition to aerobic glycolysis, TCR signaling initiates robust amino acid uptake and metabolism in T cells that strengthen mTOR activity and instruct the Th cell differentiation program [21–26]. These findings suggest that mTOR and AMPK antagonize each other to instruct T cell activation and differentiation.

#### Metabolites serve as messengers to govern T cell effector functions

Despite aerobic glycolysis and amino acid uptake being critical for T cell activation, expansion and differentiation, T cells are able to survive in glucose-depleted conditions using mitochondrial OXOPHOS activity to support their energy demand [27]. However, as a trade-off for metabolic adaptation, T cells decrease the production of effector molecules, including as IFNγ, CD40L, and IL-2. Declined mTOR activity contributes to decreased effector functions; however, several new studies have uncovered that weakened generation of glycolytic metabolites also contribute to T cell dysfunction. Production of glyceraldehyde-3-phosphate (G3P) relieves the translational restraint of IFNγ and IL-2 imposed by glyceraldehyde-3-phosphate dehydrogenase (GAPDH) [28]. Moreover, accumulation of phosphoenolpyruvate in T cells suppresses ER calcium reuptake sustaining the Ca^2+^-NFAT pathway, which controls effector molecule production [29]. These studies provide evidence that glycolysis not only provide precursors of biomass and ATP, but also allows activated T cells to sustain effector functions through both transcriptional and translational regulations.

### Metabolic pathways govern macrophage polarization

#### Metabolic preference of M1- and M2-like macrophages

**Fig. 2:**
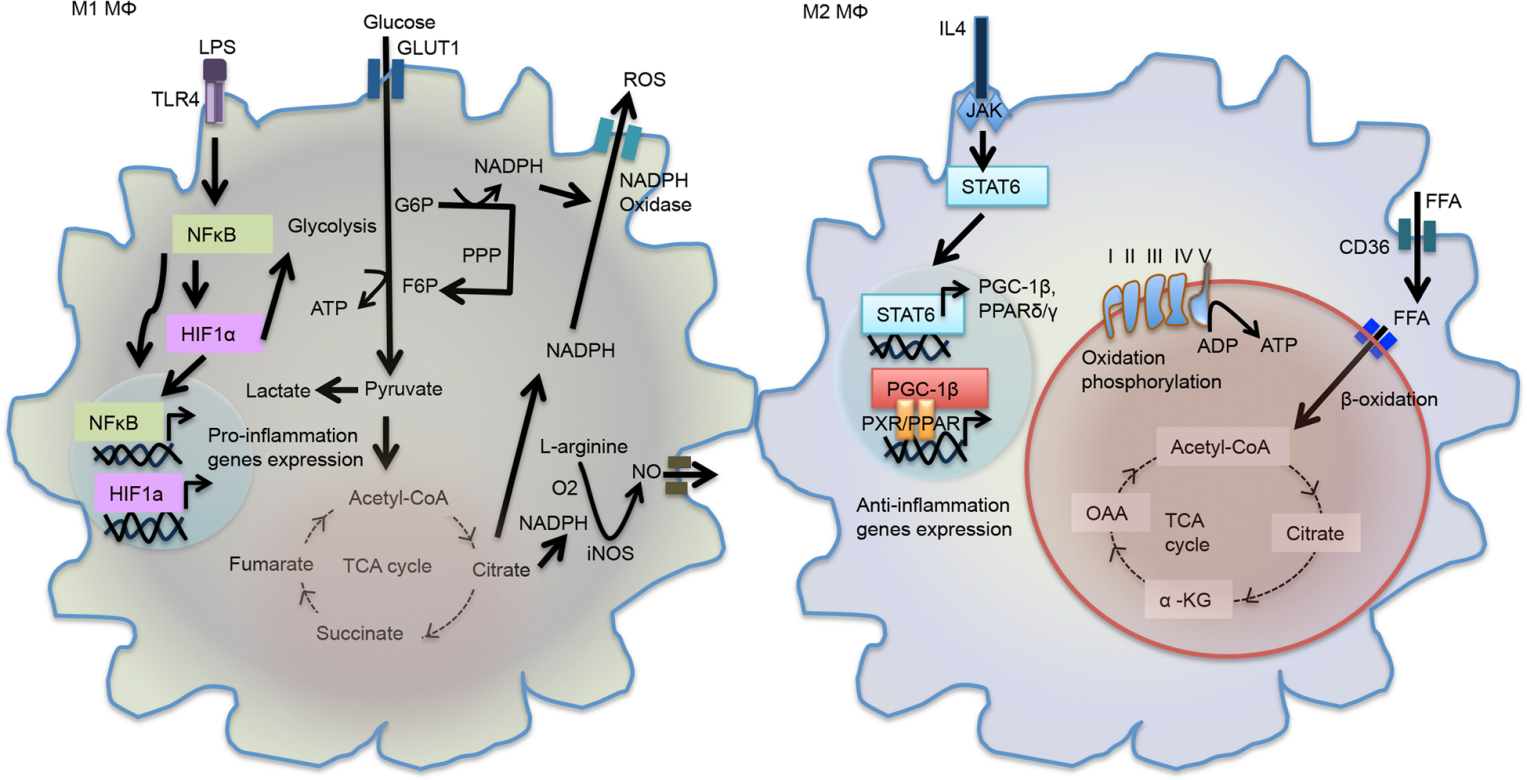
Metabolic reprogramming in M1 and M2 macrophage. LPS-induced M1 macrophages are inflammatory and generate pro-inflammatory cytokine by activation NF-κB and HIFα. M1-like macrophages display increased glycolysis to product lactate and decreased mitochondrial OXPHOS activity. M1-like macrophages also induce PPP to increase generation of NADPH for ROS production and NO synthesis. In contrast, IL4-polarized M2 macrophages activate STAT6 to induce PGC-1β and PPARδ/γ, which promote the expression of anti-inflammatory and metabolic genes. Thus, M2-like macrophages exhibit increased high rates of FAO and mitochondrial OXPHOS activity

Macrophages (MΦs) are terminally differentiated immune cells that possess high secretory, phagocytic, and antigen-presenting abilities. These activities are tightly controlled by a variety of immune stimuli such as cytokines, ligands of toll-like receptors (TLRs) and other pattern recognition receptors [30]. Resting MΦs encountering microbial stimuli, such as lipopolysaccharide (LPS) or LPS with pro-inflammatory cytokines elevate their phagocytic activity and gain a pro-inflammatory phenotype through the classical “M1-like MΦs” polarization. In contrast, MΦs can engage an alternative activation to become “M2-like”; thereby, sustaining tissue repair, angiogenesis, and Th2 immunity upon IL-4 activation. Importantly, M2-like MΦs produce more anti-inflammatory cytokines and less co-stimulatory proteins to T cells as well as demonstrating a reduced antigen-presenting ability [31]. In addition to defining stimuli and downstream signaling pathways that activate MΦs, recent research has revealed that MΦs must engage the proper metabolic switch when activated with LPS or IL-4. Specifically, TLR-induced signaling promotes MΦs aerobic glycolysis by stabilizing hypoxia-inducible factor 1a (HIF1α) and boosting mTOR activity. This increases glycolytic activity, as well as the pentose phosphate pathway (PPP) and *de novo* fatty acid synthesis (Fig. 2) [6, 30, 32]. Inhibition of glycolysis or deletion of GLUT1 prevents M1 polarization. This is phenotypically similar to treatment with either a mTOR inhibitor or AMPK activator. Moreover, it is critical that M1-like MΦs reduce mitochondrial OXOPHOS activity by suppressing succinate catabolism in order to maintain their pro-inflammatory properties [33]. In contract to M1-like MΦs, M2-like MΦs display low glucose flux but high rates of FAO and OXOPHOS via a PGC1β–dependent metabolic switch [6, 34, 35]. Of note, impairment of cholesterol efflux in MΦs results in a higher lipid content accompanying a M2-polarized phenotype [36, 37]. However, elevating lipid content with modified low-density lipoprotein (LDL) has also been shown to promote inflammatory activity associated with the M1 phenotype [38]. The underlying mechanisms on this discrepancy remain unclear, but these studies suggest that through modulating nuclear receptor activities, such as PPAR and LXR, the composition of lipid species may influence MΦ polarization [7].

#### Macrophages: double-edge swords in tumor progression and regression

The ability to use immunotherapy to exploit the host immunity to combat malignancy represents a breakthrough for cancer treatment. However, the establishment of the immunosuppressive tumor microenvironment is a major impediment for current immunotherapies [8, 39]. One of the key processes disarming anti-tumor immunity is the accumulation of M2-like tumor-associated MΦs. Tumor outgrowth is facilitated by the accumulation of M2-like MΦ via the prevention of type I immune responses elicited by T cells, the formation of abnormal vasculature and facilitating the dissipation of metastatic cancer cells [2]. However, “re-educating” M2-like MΦs to polarize into M1-like MΦs has been shown to elicit tumor regression. This occurs via restoring an immunosupportive microenvironment marked by elevated phagocytic activity, stronger antigen-presenting ability and secretion of pro-inflammatory cytokines. While the contribution of tumor-associated MΦs to cancer immune evasion and regression is established, many significant questions remain. Two major questions are how cancer cells sustain the pro-tumorigenic phenotypes of MΦs and whether cancer immunotherapy can “re-educate” tumor infiltrating myeloid cells.

### Cancer cell immune evasion is promoted via the nutrient-specialized tumor microenvironment

#### Lactic acid and glucose in the tumor microenvironment

In the past decade, it has been demonstrated that increasing aerobic glycolysis and other anabolic metabolism in cancer cells can sustain their unrestricted proliferation by generating macromolecules for biomass synthesis. Oncogenic mutations and dysfunction of tumor suppressor, such as p53, in these cancer cells contribute to their abnormal cellular energetics [3, 39, 40]. These metabolic changes are suspected to alter the nutrient composition in the tumor microenvironment. For instance, the massive generation of lactic acid from cancer cells has been shown to suppress T cell cytotoxic and effector functions; however the mechanism behind this remains unclear. Furthermore, this generation of lactic acid has also been shown to promote M2-polarization in MΦs via monocarboxylate transporter-1(MCT1)-mediated lactic acid uptake [41]. Additionally, Colegio *et. al.* discovered that lactic acid stabilizes HIF1α to drive M2-polarization [42]. Of note, in contrast to M2-polarization, several other reports have shown that HIF1α stabilization promotes a M1-like macrophage polarization [33, 43, 44]. Although it is unclear how lactic acid acts differentially trigger macrophage polarization via HIF1α stabilization, it has been reported in cancer cells that lactic acid stabilizes HIF1α in cells relying on OXOPHOS but not in cells engaging aerobic glycolysis [45]. Therefore, it is likely that the metabolic states of MΦs can coordinate with lactic acid uptake to determine their polarization fate.

Warburg glycolysis allows cancer cells to consume glucose and increase lactic acid production. Additionally, two recent studies have independently demonstrated that the tumor microenvironment is glucose-depleted contributing to diminished T cell anti-tumor responses [29, 46]. These two studies suggested that cancer cells with higher glycolytic activity have a strong capacity to evade immunosurveillance. Moreover, Ho et. al. discovered that tumor-reactive T cells can be metabolically rewired through phosphoenolpyruvate carboxykinase 1 (PCK1) overexpression. This restores the T cell anti-tumor responses when infiltrating into the glucose-deprived tumor microenvironment through regaining phosphoenolpyruavte production [29]. Moreover, Chang et. al. showed that PD-1 inhibition boosts T cell anti-tumor responses by restoring mTOR activity and glucose flux in tumor-reactive T cells. This has also been observed in the chronic viral infection model and in vitro assays [47–49]. Together, these studies provide proof-of-concept evidence that metabolic competition for nutrients in the tumor microenvironment is involved in establishing and maintaining an immunosuppressive tumor microenvironment. Furthermore, these demonstrate that restoring metabolic fitness in T cells is a promising approach to regain effective anti-tumor responses.

#### The role of amino acid and fatty acid metabolism in immunosuppressive tumors

In addition to lactic acid production and glucose metabolism, MDSCs, M2-like MΦs and cancer cells, also affect the tumor microenvironment through degradation of extracellular arginine levels via arginase 1 (Arg1) expression [42, 50]. Moreover, tryptophan metabolism in tumors has recently gained attention for how it contributes to immunosuppression. Expression of indoleamine 2,3-dioxygenase (IDO) in antigen presenting cells (APCs) and cancer cells, promotes tumor progression and associates with poor responses to cancer immunotherapies in both clinical reports and murine cancer models [26, 51]. IDO is a metabolic enzyme that degrades tryptophan to kynurenine. Upregulated IDO activity suppresses T cell proliferation by restricting tryptophan to infiltrating T cells. Moreover, the generation of kynurenine may enhance Tregs formation in tumors as it has been shown to disarm gut and neuroinflammation in other disease models [52, 53]. Overall, these findings indicate that the declined amino acid availability in tumors can be harmful for mounting effective T cell anti-tumor immunity.

As well as consuming nutrients, cancer cells also produce massive amounts of fatty acids via *de novo* fatty acid synthesis, which correlates with more invasive cancer cells [54, 55]. In addition, accumulation of adipocytes and adipocyte-like fibroblasts in tumors can contribute to their metastasis [56]. These findings indicate that the tumor microenvironment may be lipid-enriched. In support of this, tumor infiltrating MDSCs and dendritic cells displayed higher lipid contents that associate with strong immunosuppressive activity and weak antigen presentation, respectively [57–60]. Of note, a fatty acid enriched microenvironment may impinge effector T cell functions and M1-polarization in MΦs while favoring the generation of Tregs and M2-like MΦs [5, 34]. Further investigation is critical to determine whether the fatty acid content in tumors and *de novo* fatty acid synthesis in cancer cells suppress T cell anti-tumor immunity and macrophage activation.

### Unresolved questions and future directions: harnessing metabolic regulation in cancer immunotherapy

#### Metabolic states of tumor infiltrating lymphocytes and tumor-associated MΦs

Although metabolic pathways and their intermediates control immune responses in T cells and MΦs, much of our understanding has been formed based on *in vitro* cultures. These cultures generally have a defined nutrient composition and a fixed duration of stimulation. However, the tumor microenvironment *in vivo* has multiple layers of regulation. For example, the abnormal vasculature and complicated composition of cell types likely all influence the metabolic pathways and nutrients used by tumor infiltrating lymphocytes and tumor-associated MΦs. Therefore, in order to define which metabolic pathways link to effective anti-tumor responses it will be critical to determine the metabolic signature and profile in tumor infiltrating lymphocytes and tumor-associated MΦs, from progressed and regressed tumors. Additionally, metabolic adaption allows T cells to engage OXOPHOS metabolism facilitating their survival. This process requires the electron transport chain and functional mitochondria in T cells. However, it remains unclear if tumor infiltrating lymphocytes are capable of maintaining their mitochondrial fitness in order to adapt to the glucose-depleted tumor microenvironment. Thus, it will be important to examine the mitochondrial health of tumor infiltrating lymphocytes.

Metabolic competition has clearly been shown to contribute to the formation of the immunosuppressive tumor microenvironment. Therefore, it will be vital for cancer immunotherapies to develop new strategies to allow immune cells to acquire sufficient nutrients or metabolites to preserve their anti-tumor responses. Thus, examining how to combine cancer immunotherapies with metabolic inhibitors in order to sustain the metabolic fitness of tumor-reactive T cells and M1-like MΦs will be crucial. In addition, it will also be necessary to elucidate if the therapeutic efficacy of cancer immunotherapies is associated with, as yet undefined, metabolic features in tumors. A better understanding of how immunotherapies respond to different metabolic features in tumors will further improve how we apply cancer immunotherapies in patients.

#### Why does hypoxia in tumors not improve anti-tumor responses?

Hypoxia is a general feature of established tumors and contributes to elevated aerobic glycolysis by stabilizing HIF1α. Interestingly, overexpressing a degradation-resistant mutant of HIF1α or abolishing the HIF1α-degrading machinery promotes anti-tumor responses in T cells, including cytotoxic activity and production of IFNγ [61]. However, tumor infiltrating T cells fail to maintain anti-tumor responses in tumors where hypoxia is a general environmental stress. This raises an interesting question: why does hypoxia fail to boost T cell anti-tumor responses in tumors. The degree of HIF1α stabilization in cancer cells is associated with the metabolic pathways [45]. Therefore, we postulate that hypoxia fails to stabilize HIF1α in tumor infiltrating T cells due to their higher OXOPHOS metabolism, triggered by PD1 signaling, and the nutrient composition in the tumor microenvironment. If this is true, PD1 inhibition will restore hypoxia-induced HIF1α stabilization, thereby enhancing tumoricidal activity of tumor infiltrating T cells. Another possibility is that hypoxia truly boosts glycolytic rates in tumor-infiltrating T cells by stabilizing HIF1α; however, the available glucose in the tumor microenvironment is insufficient to support T cell anti-tumor responses. More extensive studies are needed to resolve this and will offer an immediate impact on the development of new combined treatments.

#### How can we improve the metabolic fitness of T cells in ACT therapy?

Reduced expression of tumor antigens and the MHCI complex in cancer cells provides a growth advantage for aggressive tumors by evading T cell mediated immune destruction. The invention of CAR T-cells gives the ability to re-direct the targeting specificity to cancer cells, thereby enhancing the anti-tumor responses in ACT immunotherapy [62]. However, the therapeutic outcome of CAR T-cells on treating solid tumors is disappointing. Interestingly, combining CAR T-cells with PD-1 blockade drastically improves anti-tumor responses, suggesting that restrictions due to the tumor microenvironment limit the CAR T cell induced anti-tumor immunity [63]. Engaging aerobic glycolysis and amino acid metabolism is a prerequisite for proper T cell activation upon TCR stimulation. However, the intracellular signaling pathways and metabolic requirement for sustaining CAR T-cell activation and anti-tumor responses are not fully understood. Given that metabolic competition between cancer cells and infiltrating T cells leads to T cell anergy and dysfunction, it will be important to investigate whether CAR signaling also relies on the metabolic switch to sustain anti-tumor responses. If it is true, it may be possible to improve the metabolic fitness and flexibility of CAR T cells with genetic and chemical approaches.

In addition to CAR T-cells, memory tumor-reactive T cells have been shown to elicit superior anti-tumor responses than effector T cells do [64]. During *in vitro* assays secondary effector T cells generated from memory T cells, display stronger aerobic glycolysis and IFNγ production than primary effector T cells [65, 66]. Therefore, these secondary effector T cells can plausibly acquire sufficient glucose when in the tumor microenvironment even under “metabolic competition” with cancer cells. Moreover, memory T cells also possess healthier mitochondria, which may help adaptation to a nutrient crisis more effectively [65]. Accordingly, it will be important to gain a detailed understanding of the underlying mechanisms contributing to the stronger metabolic fitness seen in secondary effector T cells. This knowledge will feasibly provide a blueprint for manipulating transferred tumor-reactive T cells. Ultimately, this may allow tumor-reactive T cells to be conferred with enhanced metabolic fitness and effector functions specifically in the nutrient-specialized tumor microenvironment.

## Conclusions

**Fig. 3:**
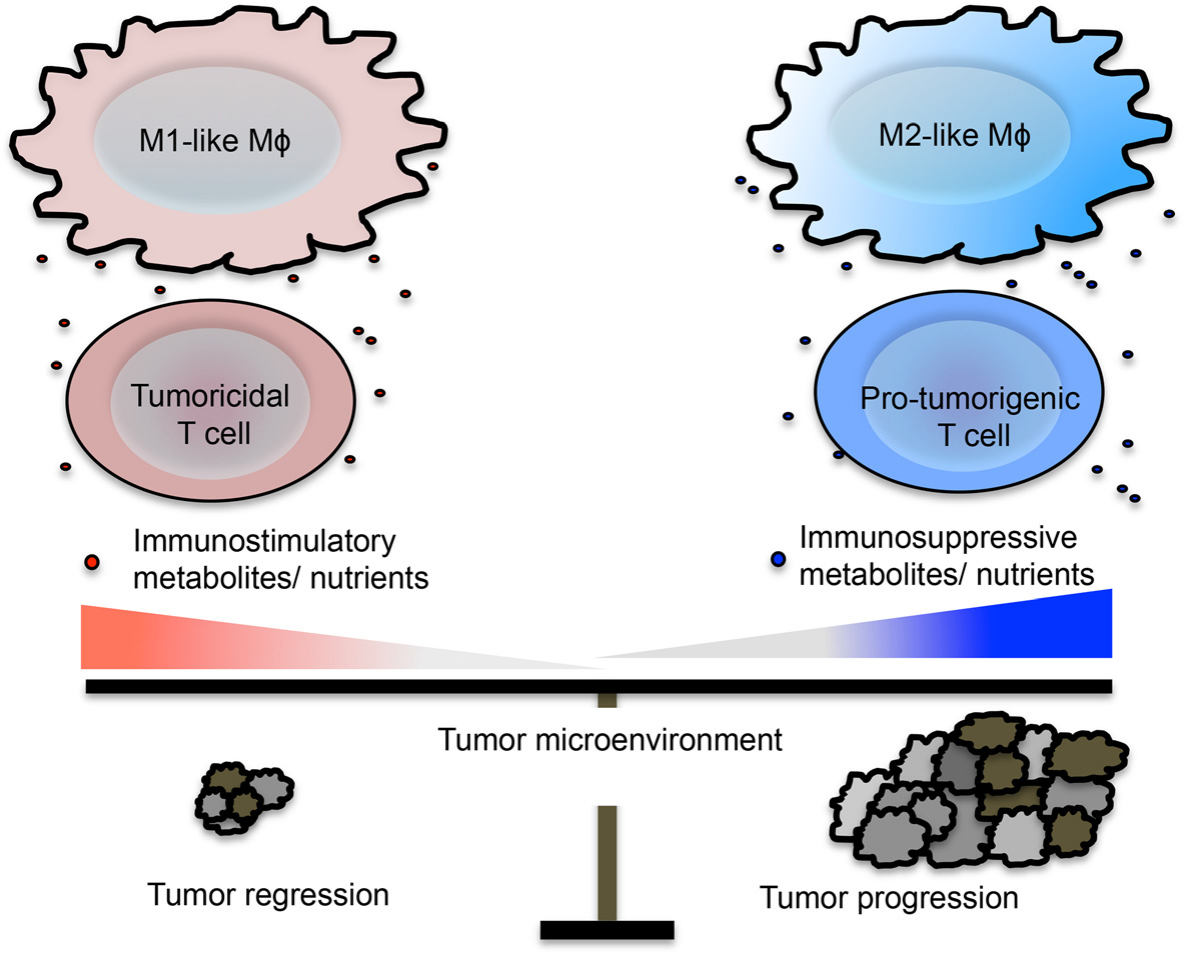
Environmental nutrients control immune cells phenotype. The composition of nutrients within tumor environment affects metabolic fitness of infiltrating immune cells. Thus, new approaches to rewire metabolic fitness in tumor infiltrating immune cells might boost immune response in conjunction with current cancer immunotherapies

Cancer immunotherapy provides an exciting opportunity to introduce a powerful weapon to the arsenal of cancer treatment. However, it is crucial to understand how the intensive communication in the tumor microenvironment efficiently diminishes anti-tumor immunity. Modulation of cellular metabolism has been shown to play a cardinal role in controlling immune responses in a variety of immune cells. Despite this, deregulated cancer metabolism represents a newly appreciated hallmark for cancer cells. Further research in this field will be vital to provide the foundation for new treatments that target cancer metabolism. Here, we hypothesize that changing the immunostimulatory and immunosuppressive nutrients and metabolites within the tumor microenvironment denotes a new immunomodulatory mechanism in tumors (Fig. 3). Altering the nutrient composition allows cancer cells to evade from immunosurveillance and, even coax the formation and accumulation of pro-tumorigenic immune cells. A clear understanding of how the immunometabolic regulation controls immune responses, as well as identifying immunostimulatory and immunosuppressive nutrients, will allow immune responses in tumors to be rebalanced with genetic and/or nutritional approaches.

## Abbreviations

ACT: Adoptive cell transfer
DC: Dendritic cells
CAR: Chimeric antigen receptor
APC: Antigen-presenting cell
OXOPHOS: Oxidative phosphorylation
